# Transcriptional analysis of an immune-responsive serine protease from Indian malarial vector, *Anopheles culicifacies*

**DOI:** 10.1186/1471-2199-8-33

**Published:** 2007-05-15

**Authors:** Janneth Rodrigues, Neema Agrawal, Anil Sharma, Pawan Malhotra, Tridibes Adak, Virander S Chauhan, Raj K Bhatnagar

**Affiliations:** 1 Insect Resistance Group, International Centre for Genetic Engineering and Biotechnology (ICGEB) PO Box 10504, Aruna Asaf Ali Marg, New Delhi 110067, India; 2 Malaria Group, ICGEB, New Delhi 67, India; 3 National Institute of Malaria Research 2, Nanak Enclave, (Radio Colony), Delhi 110009, India; 4 Laboratory of Malaria and Vector Research, National Institute of Allergy and Infectious Diseases, NIH, Bethesda, Maryland, USA

## Abstract

**Background:**

The main vector for transmission of malaria in India is the *Anopheles culicifacies *mosquito species, a naturally selected subgroup of which is completely refractory (R) to transmission of the malaria parasite, *Plasmodium vivax*;

**Results:**

Here, we report the molecular characterization of a serine protease (*acsp30*)-encoding gene from *A. culicifacies*, which was expressed in high abundance in the refractory strain compared to the susceptible (S) strain. The transcriptional upregulation of *acsp30 *upon *Plasmodium *challenge in the refractory strain coincided with ookinete invasion of mosquito midgut. Gene organization and primary sequence of *acsp30 *were identical in the R and S strains suggesting a divergent regulatory status of *acsp30 *in these strains. To examine this further, the upstream regulatory sequences of *acsp30 *were isolated, cloned and evaluated for the presence of promoter activity. The 702 bp upstream region of *acsp30 *from the two strains revealed sequence divergence. The promoter activity measured by luciferase-based reporter assay was shown to be 1.5-fold higher in the R strain than in the S. Gel shift experiments demonstrated a differential recruitment of nuclear proteins to upstream sequences of *acsp30 *as well as a difference in the composition of nuclear proteins in the two strains, both of which might contribute to the relative abundance of *acsp30 *in the R strain;

**Conclusion:**

The specific upregulation of *acsp30 *in the R strain only in response to *Plasmodium *infection is suggestive of its role in contributing the refractory phenotype to the *A. culicifacies *mosquito population.

## Background

The *Anopheles culicifacies *mosquito is the main vector of the human malaria parasite, *P. vivax*, in the Indian sub-continent and is responsible for approximately 65% of new malaria cases annually [1]. The natural transmission cycle of *Plasmodium *parasite requires successful completion of a complex sporogonic cycle in the midgut and the salivary glands of the *Anopheles *mosquito, a process that takes place over a period of two weeks. This developmental cycle can be blocked by the innate immune responses of the mosquito thereby resulting in the elimination of pathogen in the vector itself [2].

Mosquito vectors differ in their efficiency of transmitting malaria; some are refractory and completely block transmission of parasite [2]. Naturally evolved and genetically selected refractory strains are important for the study of mechanisms that mediate *Plasmodium *killing [3-5]. Genetically selected susceptible and refractory strains (4Ar/r and L3-5, respectively) are described in African mosquito *Anopheles gambiae *[3]. The R strain blocks parasite development by melanotically encapsulating the ookinetes after invasion of the midgut [6]. Understanding the molecular basis of such refractory phenotypes may contribute to the development of novel strategies for malaria control, which would rely on elimination of the parasite in the mosquito itself.

Recently, Adak *et al*. (2006) reported the isolation of a naturally occurring field strain of *A. culicifacies *that is 100% refractory to *P. vivax *and partially resistant to *P. falciparum *and *P. vinckei *(rodent parasite) [7,8]. In the R strain, brown capsular ookinetes characteristic of the melanization reaction were microscopically observed in the mosquito midgut within 24 hours of *Plasmodium *– infected blood feeding. Normal uncoated ookinetes were observed in the S strain indicating that strain refractoriness in *A. culicifacies *could be attributed to melanotic encapsulation of ookinetes. The melanotic capsule consists of a proteinaceous poly-quinone material surrounding the parasite and killing of the parasite may be mediated by toxic byproducts of the melanization cascade reactions, such as free radicals or by starvation within the capsule [9,10].

The melanization reaction starts with a modulatory serine protease cascade, leading to proteolytic cleavage and activation of prophenoloxidases (PPOs) to phenoloxidases (POs), the key enzymes for melanin production [11,12]. In addition, serine proteases are also involved in signaling and amplification cascades that lead to the activation of specific defense mechanisms, such as melanization, coagulation and induction of anti-microbial peptides. An increasing number of serine proteases involved in immune responses have been isolated and characterized from *A. gambiae*. The majority of these serine proteases are present in the hemolymph and expressed by hemocytes. In *A. gambiae, CLIPB9, CLIPB4 and CLIPB1 *serine proteases are induced during immune responses and have sequences characteristic of prophenoloxidase activating enzymes [13-17]. The role of several of these proteases in melanization and killing of parasites has been examined by RNA interference [18-20].

In the present study, we report the isolation of a serine protease (*acsp30*) from the body tissue of *A. culicifacies*. *Acsp30 *was poorly transcribed in the S strain whereas abundant transcript was observed in the R strain. Expression of *acsp30 *was constitutive in both the strains but was strongly upregulated upon parasite invasion in the R strain. Although, the nucleotide sequence of *acsp30 *cDNA and genomic clone were identical in R and S strains, a significant sequence divergence of upstream regions was observed. The promoter in R and S strains was localized within -702 bp of the translation initiation site; quantification of promoter strength by the luciferase-based reporter gene assay revealed a 1.5-fold higher activity in the R strain compared to the S strain. Our studies suggest divergent promoter sequences recruit differential transcription factors resulting in a varied expression of *acsp30 *in the R and S strains. In addition, differential distribution of nuclear proteins in the two strains could also contribute to disparity in *acsp30 *transcription.

## Results

### Cloning and analysis of *acsp30 *of *A. culicifacies*

**Figure 1:**
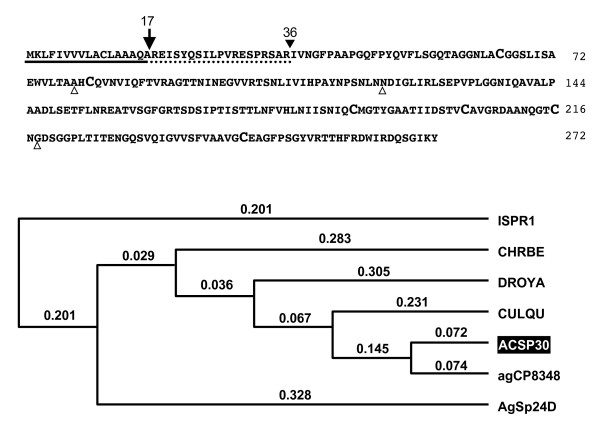
**(A) Deduced amino acid sequence of ACSP30 from *A. culicifacies***. The 816 bp cDNA encoded a 272 amino acid protein. The three residues of the catalytic triad are marked by 'Δ'. The putative 17 amino acid signal peptide is underlined with a solid line and the putative 19 amino acid propeptide is underlined with a dotted line. '' indicates the cleavage site of signal peptide and '▼' shows the activation site. The conserved cysteines are shown in bold and larger font. **(B) Phylogenetic tree describing the sequence similarity of insect serine proteases**. The tree was constructed by the neighbour-joining method with the Best Tree mode. A value of 0.1 corresponds to a difference of 10% between two sequences. GenBank™ accession numbers are as follows; *A. gambiae *predicted serine protease (agCP8348), AAAB01008980.1; *A. gambiae *serine protease (AgSp24D), U21917: *A. gambiae *immune-responsive chymotrypsin-like serine protease-related protein (ISPR1), AF203336: *A. gambiae *EASTER-like serine protease (Sp14D1), AF007166: *Culex quinquefasciatus *serine protease (CULQU), Q23731: *Chrysomya bezziana *serine protease K17/F1R1 (CHRBE), Q9GSM5; *Drosophila yakuba *serine protease (DROYA), Q6X139.

A PCR-based strategy was employed to isolate the serine protease-encoding gene (*acsp30*) from the body tissue (thorax and abdomen) of *A. culicifacies*. The 816 bp full-length cDNA encoded a 272 amino acid protein, ACSP30, corresponding to molecular mass of approximately 30 kDa. Computer-assisted analysis revealed that the first 17 amino acids of ACSP30 constituted a signal peptide and the next 19 form a propeptide. Mature ACSP30, 236 amino acids in length possessed structural elements characteristic of all serine proteases such as the conserved catalytic triad (HDS) and six cysteine residues (Fig. 1A).

By using the ClustalW alignment program (Mac Vector version 7.0), the deduced amino acid sequence of ACSP30 was aligned with reported sequences from other insects. ACSP30 showed highest sequence similarity (83%) with a predicted serine protease (Protein Accession: EAA13956.1) from *A. gambiae *genome (ENSANGP00000014448). Amongst the reported *A. gambiae *serine proteases, the ACSP30 showed maximum similarity with AgSp24D (31%) (Fig. 1B) [21]. ACSP30 also shared sequence similarity with serine proteases from *Chrysomia bezziana *(Screwworm fly) (27%), *Drosophila yakuba *(26%) and *Culex quinquefasciatus *(30%) which are reported to function in dissolution of fibrin of blood clots and as proteolytic factors in a variety of processes including embryonic development, tissue remodeling, tumor invasion and inflammation [22]. Interestingly, ACSP30 shared low (<15%) sequence homology with the Clip-domain serine proteases (CLIPs) that are implicated in defense mechanisms in insects [18,19,23-26].

### Differential expression of serine protease in R and S strains

**Figure 2:**
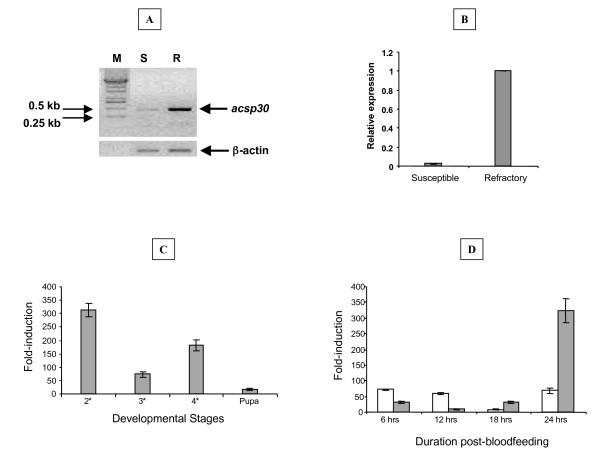
**Expression pattern of *acsp30 *in *A. culicifacies *female mosquito**. Determination of relative transcript abundance of *ascp30 *in R and S strain adult mosquitoes by semi-quantitative RT-PCR **(A) **and real-time PCR **(B)**. **(C) **Expression of *acsp30 *at various stages of mosquito development, second instar (2*), third instar (3*), fourth instar (4*) and pupa, is depicted as fold-induction over levels present in naïve adult female mosquitoes by real-time PCR. **(D) **Temporal induction of *acsp30 *upon *Plasmodium *infection. Refractory mosquitoes were fed on uninfected blood (open bars) and on *P. vinckei *infected blood (shaded bars) and transcript levels of *acsp30 *were measured by real-time PCR at different time intervals post-blood meal (PBM). For all realtime RT-PCR experiments, RNA isolated from body tissue (abdomen and thorax) of mosquitoes was used as template and expression levels of *acsp30 *were measured by using the Comparative C_T _Method. Transcript levels were normalized to the internal control, *β-actin *and shown as fold induction relative to the naïve adult female mosquitoes. Representative data (mean ± S.D.) from three independent experiments are shown.

The relative expression level of *acsp30 *transcript in five-day old naïve adult female mosquitoes of the R and S phenotypes was analyzed by semi-quantitative RT-PCR (Fig. 2A) and real-time PCR (Fig. 2B) using β-actin as an internal control. Though *acsp30 *was expressed constitutively in the R and S strains, there was a significant difference in their expression levels. In naïve adult female mosquitoes, transcript levels of *acsp30 *were 40-fold higher in the R strain compared to S. *Acsp30 *was expressed constitutively during development of mosquito; a varying pattern of expression was observed from second instar larvae to pupa. A dramatic 300-fold increase in *acsp30 *transcript was observed in the second instar larvae as compared to the levels present in the adult mosquito (Fig. 2C).

### Immune responses to *Plasmodium *infection

Both refractory and susceptible 4–6 day old adult female mosquitoes were separately fed on blood of Balb/c mice infected with *Plasmodium vinckei petteri*. The refractory *A. culicifacies *mosquito strain is partially resistant to the rodent malarial parasite [7]. Mosquitoes fed on blood of uninfected mice were included as blood-fed controls. Temporal expression of *acsp30 *was monitored by real-time PCR at regular intervals after feeding (Fig. 2D). In the R strain, a 68-fold increase in *acsp30 *transcript level was observed 24 hours after feeding on uninfected blood. Mosquitoes fed with infected blood exhibited a 300-fold increase in the transcript levels over the naïve unfed female mosquitoes. The 24 hour post-feeding upregulation of *acsp30 *expression coincided with microscopic observation of encapsulated *Plasmodium *ookinetes in the midgut of the R strain. In contrast, the expression levels of the *acsp30 *remained unaltered upon feeding uninfected or parasite-infected blood in the S strain (data not shown).

### Structural analysis of *acsp30 *upstream regulatory sequences from R and S strains

The cDNA corresponding to the *acsp30 *was cloned from both R and S strains and sequenced. A comparison of the two sequences did not reveal any difference between the two strains. Since the cDNA sequence of *acsp30 *was identical in both the strains, we isolated the corresponding genomic DNA using PCR. The 887 bp gDNA fragment was slightly bigger than the cDNA indicating the presence of an intron. Sequence analysis revealed that the gene has a single intron of 71 bp at its 5' end and is a phase 0 intron. Since the location and the sequence of the intron were identical in both the strains, the upstream sequences of *acsp30 *were isolated and evaluated for promoter activity.

**Figure 3:**
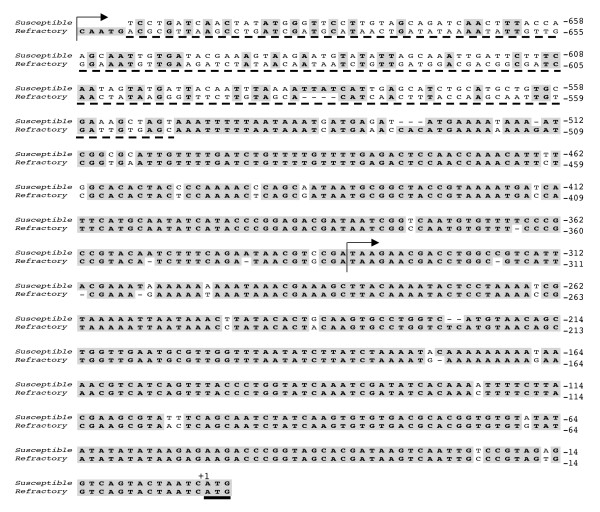
**Computer-assisted analysis of *acsp30 *upstream sequences from refractory and susceptible strains of *A. culicifacies***. **(A) **Alignment of the two sequences. The translational start site (ATG) is underlined and the numbers to the right denote the positions of the adjacent nucleotide in each line, relative to ATG at +1. The arrows indicate the boundary of the 333 bp and 702 bp constructs used for promoter analysis. The sequence marked with a dashed line represents the region of maximum dissimilarity between R and S strains. **(B) **Comparative schematic diagram depicting the features of the upstream regions. Dotted line delineates the region displaying equal promoter activity and solid line delineates the region showing higher promoter activity in the R strain than in the S. (**C and D**) Organization of the upstream regulatory sequences of *acsp30 *in the S (Fig. 3C) and R (Fig. 3D) strain mosquitoes. Two TATA like boxes are present with corresponding arthropod initiator (Inr) sequences and capsites, which constitute the core promoter. Vertebrate upstream immune response elements are represented in bold fonts and underlined; hepatic nuclear factor 5 (HNF-5), nuclear factor interleukin 6 (NF-IL6), interferon consensus response elements (ICRE) and nuclear factor endothelial adhesion molecules (NF-ELAM 1), ecdysteroid response elements (EcRE) and the different GATA factors are in bold face and italicized. Consensus sequences for all these motifs are described in the text. Insect regulatory sequence motifs are boxed. The numbers to the left denote the positions of the first nucleotide in each line, relative to ATG at +1. The open block arrow indicates the predicted transcriptional start site (TSS).

Using a PCR-based directional genome walking protocol, regions upstream to *ascp30 *were cloned from R and S strains. Amplicons of 1.4 kb and 0.7 kb were obtained in the R and S strains respectively. Upstream sequences (702 bases) from the R and S strains were aligned by ClustalW using MacVector (Version 7.0) and EMBOSS-Align (Fig. 3A). A high degree of sequence similarity (94.2%) was observed up to 333 bp, upstream of the translational start site (ATG). Beyond this region there was a considerable divergence in the gene sequence. The region between -702 bp and -333 bp showed 54% similarity with 41.7% gaps and only 30% similarity was observed in the region between -702 bp and -500 bp with 65.8% gaps (Fig. 3B).

*In silico *analysis of the upstream sequences of *acsp30 *revealed characteristics of RNA polymerase II-transcribed promoters. Using the computer-based promoter prediction tool, two putative transcription start sites were predicted to be located at -31 bp (score cutoff of 1.0) and -40 bp (score cutoff of 0.8) upstream of ATG in R and S strains. The region surrounding this transcription start point corresponded to the arthropod initiator sequence, which is found in the interval -10 to +10 of 25% of arthropod RNA polymerase II-transcribed promoters as described by Cherbas and Cherbas (1993) [27]. This reinforced the prediction of this site as the true transcription start site. In both strains, two TATA motifs were found at position -53 and -189 from the ATG and two arthropod transcription initiator motifs TCAGT [27] were present at position -12 and -152. Using the consensus DCAKTY [27], putative capsite was found at position -31 and -152 respectively in the R and S strains. These sites constituted the putative core promoter elements (Fig. 3C and Fig. 3D).

Regulatory sequence motifs in both the strains were searched from within the insect family using the MatInspector Program [28] and 29 consensus matches were detected in the S strain and 19 in the R strain, all of which were based on factors present in *Drosophila*. In the S strain, the 29 different matrices were well distributed along the entire 702 bp upstream region but in the R strain such motifs were absent in the region between -304 bp and -702 bp and the 19 matrices were restricted to the -1 to -303 bp region. A comparative analysis of putative binding sites is depicted in Table 1.

The upstream regulatory sequences of the R and S strains were also scanned for putative vertebrate regulatory elements. Several interesting motifs were found in these regions. Computer-assisted analysis revealed motifs which were homologous to the response elements for transcription factors described in the promoters of other insect immune-responsive genes and mammalian acute-phase protein genes and are listed in Table 2[29]. Of the several motifs listed, an interesting observation relates to the two potential regulatory motifs for the ecdysone responsive element (EcRE) (consensus RGKKSNNNGNNYK) [30]. In the R strain, EcRE are located at positions -199 to -211 bp (GGTTGAATGCGTT) and -5 to -17 bp (AGTGGTCAGTACT) on the plus strand while only one such motif was located in the S strain at a position identical to the R strain (-199 to -211 bp). At present, it is difficult to assess the consequences of these differences on the refractory phenotype, a detailed mutational and deletion analysis is necessary to study the significance of these observations.

### Functional analysis of the *acsp30 *promoter in R and S strains

**Figure 4:**
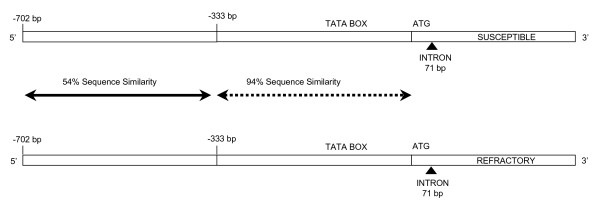
**Assessment of promoter activity of *acsp30 *from R and S strains of *A. culicifacies *using luciferase-based reporter assay in *Drosophila *S2 cell line**. Constructs, pGL3-Ref (702 bp and 333 bp) and pGL3-Sus (702 bp and 333 bp) were transfected in the *Drosophila *Schneider (S2) cell line using lipofectin and the luciferase activity measured. Promoterless vector pGL3-Basic and pGL3-Control plasmid with the SV40 promoter served as controls. Reported activities were based on three independent transfections and the data was recorded in relative light units (RLU).

Based on the homology of upstream sequences of *acsp30 *between R and S strains, two regions were selected for evaluating the promoter activity. The region spanning -1 to -333 bp showed 94% similarity and the other region spanning -1 to -702 bp showed 72% similarity. These regions from the R and S strains were cloned in promoterless pGL3-Basic vector containing firefly luciferase reporter gene and transfected into *Drosophila *S2 cells. The luciferase activity from all the four constructs was higher than that of the vector (pGL3-Basic) and control (pGL3-Control) suggesting that the upstream gene sequences from R and S strains contain regulatory elements for their respective promoters (Fig. 4). The two constructs pGL3-Ref333 and pGL3-Sus333 yielded similar levels of luciferase activity, while pGL3-Ref702 from the R strain showed 1.5-fold increase in luciferase activity compared to the S strain.

### Differential binding of nuclear proteins to *acsp30 *upstream sequences from R and S strains

**Figure 5:**
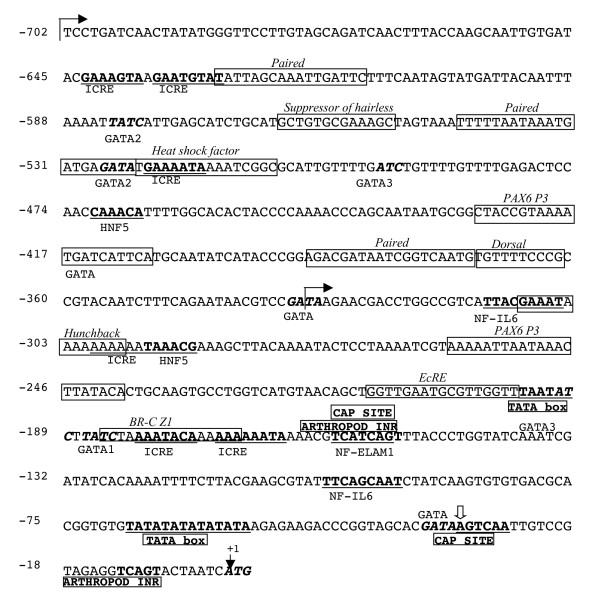
**EMSAs with *A. culicifacies *refractory (R) strain nuclear extract and 400 bp probes from upstream sequences of *acsp30 *of S and R strains**. **(A) **EMSA showing the binding pattern of nuclear proteins extracted from body tissue of five-day old adult female R strain mosquitoes when incubated with S400 (lane 2) and R400 (lane 3) probes at 37°C for 25 minutes. Free probes were run in lane 1 (S400) and lane 4 (R400). **(B) **The radiolabelled probe R400 was incubated with refractory nuclear extracts without competitor (lane 3) and in the presence of unlabeled probe at 50-fold (lane 4) and 100-fold (lane 5) molar excess. Arrows indicate the migration of complex A (slow migrating) and B (fast migrating). The intensities of the signals were quantified with respect to Phospholmager signals by the Image Analysis Software, ImageQuant TL (Amersham Biosciences) and represented as the percentage ratio of S400 to R400 signal intensities (Fig. 5A) and as percentage ratio to the uncompeted (R400) signal intensities (Fig. 5B).

**Figure 6:**
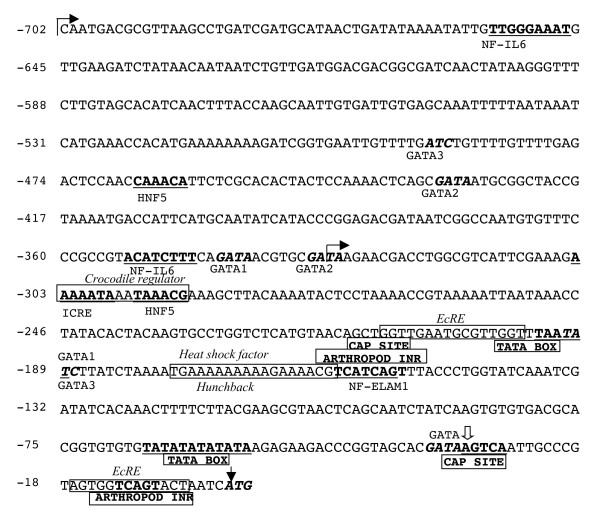
**EMSAs using nuclear extracts and 188 bp probes from *acsp30 *upstream sequences**. Nuclear proteins were extracted from body tissue of 5-day old adult female of S strain (A) and R strain (B) and incubated with S188 (lane 2) and R188 (lane 3) probes. Free probes were run in lane 1 (S188) and lane 4 (R188). Arrows indicate the migration of complex A (slow migrating) and complex B (fast migrating). The intensities of the signals were quantified with respect to Phospholmager signals by the Image Analysis Software, ImageQuant TL (Amersham Biosciences) and represented as the percentage ratio of S188 to R188 signal intensities.

**Figure 7:**
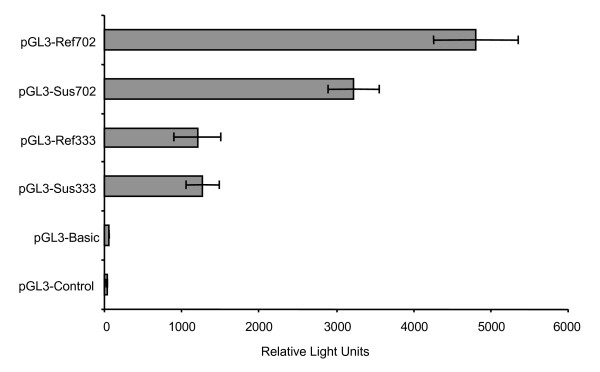
**EMSAs using nuclear extracts and 100 bp probes from *acsp30 *upstream sequences**. Nuclear proteins were extracted from body tissue of 5-day old adult female of S strain (A) and R strain (B) and were incubated with S100 (lane 2) and R100 (lane 3) probes. Free probes were run in lane 1 (S100) and lane 4 (R100). Arrows indicate the migration of complex A (slow migrating) and complex B (fast migrating). The intensities of the signals were quantified with respect to Phospholmager signals by the Image Analysis Software, ImageQuant TL (Amersham Biosciences) and represented as the percentage ratio of S100 to R100 signal intensities.

The increase in promoter activity in the R strain was attributed to sequence differences within the 400 bp region spanning -333 to -702 bp upstream of the start codon. To determine the effect of such differences on the binding of nuclear proteins, electrophoretic mobility shift assays (EMSAs) were performed. EMSA experiments with three different probes (400 bp, 188 bp and 100 bp) allowed us to determine the minimum upstream region that shows difference in binding of nuclear proteins and thus might be responsible for differential expression of *acsp30 *in the two strains. EMSAs using nuclear extracts from the R strain and R400/S400 probe revealed two complexes, a sharp slow migrating band, complex A and a faster moving diffused band, complex B (Fig. 5A, lane3). Both the complexes were observed when R188 probe was incubated with nuclear extract from R strain (Fig. 6B, lane 3). Interestingly, formation of complex B was nearly abolished on R100 probe but complex A formation remained unaffected (Fig. 7B). These results clearly indicated that the 88 bases (-602 to -514 bp) missing in R100 probe were critical for the assembly of transcription factors forming complex B but the 100 bp upstream region (-702 to -602 bp) was important and sufficient for binding of nuclear proteins forming complex A.

In general, the binding of nuclear proteins to probes derived from the S strain was less compared to that from the R strain, which further emphasized the importance of differences in upstream regions of *acsp30 *from both the strains. The greater intensity of the bands with the R probes showed that the formation of both the complexes was more on R probe than S; an approximately 25% increase in DNA binding activity of both the complexes was observed on R400 as compared to S400 probe. Importantly, when S188 was used as a probe, there was an approximately 50% reduction in the formation of complex B than on R188. This could be a consequence of an increase in sequence divergence (70%) in this region between the two strains.

Specificity of interaction of nuclear proteins with various probes from R and S strains was evaluated by competition assays in the presence of corresponding specific cold probe. The binding of nuclear factors to 400 bp upstream sequence from R strain (R400) was highly specific as the formation of complexes A and B was reduced to 25% in presence of 100-fold molar excess of unlabeled 400 bp cold probe in the EMSA binding reaction mixture (Fig. 5B). Competition experiments with sequentially shorter fragments from R and S strains also generated similar results showing the specificity of binding of nuclear factors to all the probes (data not shown).

We also performed EMSA experiments using nuclear extract from both the strains to evaluate the presence of additional transcription factors in the R strain that could be absent in the S strain. Noticeably, the binding pattern of nuclear proteins from S strain to S188 and R188 probes (Fig. 6A) were different from that of nuclear proteins from R strain. A similar result was obtained with S100 and R100 probes (Fig. 7). When a nuclear extract from the S strain was used with R100 probe, the faster migrating band, complex B did not form. This is indicative of either a lack of the transcription binding factors in the S strain that form complex B or their low concentrations that prevent detection. The association of putative binding factors was quantified by converting intensity of signals to numerical values by using the Image Analysis Software, ImageQuant TL (Amersham Biosciences) and the results were presented as bars in figures, 5, 6 and 7.

## Discussion

Naturally occurring, malaria-resistant strains, like the refractory strain *of Anopheles culicifacies *are very rare in the field. Low occurrence of such resistant phenotype is attributed to the selection pressures on the immune system that are responsible for strain survival and reproductive success. Genetic analyses of this strain have revealed that the genes for refractoriness in *A. culicifacies *are dominant and autosomal [8]. Here, we present analyses of one of the putative biochemical factors of natural population of *A. culicifacies *mosquito that displays refractoriness to *Plasmodium *by melanotic encapsulation of ookinetes. The phenoloxidase cascade that is responsible for the melanization event recruits serine protease to activate prophenoloxidase, thereby leading to the formation of phenoloxidase, tyrosine and quinones [12]. Serine proteases are reported to play a determinant role in triggering melanotic encapsulation of parasites in many malaria-refractory mosquito strains [5,18,31].

We focused our analysis on one of the early determinants of encapsulation phenotype, serine proteases. A parasite-inducible serine protease-encoding gene (*acsp30*) was isolated from the body tissue of *A. culicifacies*. The deduced protein, ACSP30, shared 30% or less sequence similarity with serine proteases from other insects such as *Drosophila, Chrysomia *and *Culex*. These serine proteases are reported to function mainly in dissolution of fibrin clots or in embryonic development. Notably, ACSP30 did not possess a CLIP domain and shared a very low sequence homology (<15%) with Clip domain serine proteases that are implicated in melanization of malaria parasites [18,20,32]. It may represent a divergent class of hitherto undescribed serine protease that plays a role in melanotic encapsulation of the parasite.

Importantly, transcriptional analysis of *acsp30 *revealed that it could play an important role in the mosquito immunity. Real time analysis demonstrated that the levels of *acsp30 *were 40-fold higher in the refractory strain as compared to the susceptible strain even in the absence of any challenge. The strain seems to have evolved the capacity to constitutively express high levels of serine protease, which could serve as a molecular marker for differentiating R and S strains. The high transcript levels of *acsp30 *in the R strain prompted us to further investigate its role inresponse to parasite invasion. In this regard, time-dependent experiments that involved feeding R and S mosquitoes uninfected and *Plasmodium*-infected blood were carried out. Expression levels of *acsp30 *were significantly increased upon un-infected blood feeding (68-fold over the naïve unfed control) in the R strain but no such induction was observed in the S strain, thereby making it a strain-specific response. Upon feeding parasite-infected blood, the transcription of *acsp30 *was further upregulated (4.4-fold over the uninfected blood-fed control) suggesting that endogenous levels of the transcript were not sufficient to combat the invading parasite and an added induction was required to block parasite development. Noticeably, upregulation of *acsp30 *transcript levels 24 hours after parasite-infected blood feeding coincided well with the appearance of melanotically encapsulated ookinetes in midgut of the R strain of *A. culicifacies*.

Both, time and high level of induction of *acsp30 *are suggestive of its role in PPO-mediated melanization cascade triggered in response to *Plasmodium*. Importantly, the transcript levels of *acsp30 *were unaffected when the non-melanizing S strain was fed on *Plasmodium*-infected blood (data not shown). This result further strengthened the possibility of involvement of *acsp30 *in contributing towards the refractory phenotype to the mosquito along with other genes. Interestingly, when the R strain of *A. culicifacies *was challenged with a gram-positive bacterium, *Micrococcus luteus *(data not shown), the expression levels of *acsp30 *were not affected, indicating that the induction of *acsp30 *was a *Plasmodium*-specific response.

Recently, gene silencing of Clip domain serine proteases by RNAi have revealed their involvement in killing of parasite and melanization of sephadex beads [18-20]. The loss of refractory phenotype of the R strain mosquitoes upon injection of dsRNA corresponding to *acsp30 *could illuminate the specific role of ACSP30 in melanization. The steps that lead from upregulation of *acsp30 *to encapsulation in the refractory strain are not clear at present. It is possible that upregulation may be a postmelanotic event facilitating clearance of parasite [20].

To understand the basis of differential expression of *acsp30 *in the R and S strains, structural analysis of the gene was carried out. Genomic DNA and cDNA sequences of *acsp30 *from both strains were found to be identical, including the presence of a 71 bp intron. Therefore, we concluded that the coding and the non-coding regions of the gene were not responsible for the observed differential expression in the two strains. The upstream sequences of the gene from both the strains were cloned, scanned and evaluated for promoter activity using a promoterless, luciferase reporter vector. The promoter activity of the 333 bp regions was nearly identical whereas 702 bp region from the R strain yielded 1.5-fold higher luciferase activity than the S strain. This observation suggested that the sequence between -702 bp and -333 bp was responsible for the differences in promoter activity in R and S strains. Sequence alignment of the 400 bp region (-333 to -702 bp) from both strains revealed 54% similarity compared to 94% similarity in the 333 bp region (-1 to -333 bp) thereby providing support to above observation.

An *in silico *analysis of *acsp30 *upstream sequences was carried out using the MatInspector program. It is premature to identify amongst the listed transcription factors any correlative specifics for encapsulation, nevertheless, one particular transcription factor that deserves special mention is Dorsal. It is mainly engaged in transcriptional activation of genes involved in dorsoventral patterning [33]. The mosquito orthologue of dorsal is Gambifl (REL1), which has been shown to translocate to the nucleus following bacterial but not *Plasmodium *infection [34]. Interestingly, this site was present in the upstream sequences of *acsp30 *from susceptible but not from the refractory strain.

Several signature sequence motifs implicated in promoters of immune-responsive genes from *A. gambiae *and *Aedes aegypti *[29,35-37] were also present within the upstream sequences of *acsp30*. Notably, the consensus for NF-κB, which is reported in *Aedes *[35] and *A. gambiae *[29] defensin genes is absent in *acsp30*, suggesting that defensin pathway-associated serine protease is different from the phenoloxidase-associated serine protease. Interestingly, the *A. gambiae *PPO1 gene that codes for melanin-synthesizing enzyme prophenoloxidase is the first mosquito immune gene reported to contain two ecdysteroid response elements (EcRE's) in its promoter sequence [38]. Using reported consensus sequences, two putative EcREs were located in *acsp30 *upstream sequences from the R strain and one in the S strain [30]. The significance of these differences and their eventual effect on refractoriness in *A. culicifacies *remains to be ascertained. A closer look at the nucleotide composition of the upstream sequences in the two strains revealed a very high G+C content in the R sequences and high A+T in the S sequences which probably could influence the affinities or the strength of the DNA-protein interactions.

EMSA experiments were carried out to further characterize the upstream sequences of *acsp30 *from both strains. Together with 400 bp, which showed higher promoter activity in R strain, two more regions within (100 and 188 bp) were chosen for EMSA experiments. Nuclear proteins from the R strain mosquitoes interacted with R400 probe to form two complexes, larger complex A and smaller complex B. The complex A appeared as a discrete band whereas complex B was observed as a diffused band in the agarose gel with greater intensity than complex A. This could be due to greater affinity of complex B for the probe than complex A or due to its higher abundance. The sequence differences between R400 and S400 led to a 25% reduction in formation of both the complexes on S400, showing an overall decrease in number of transcription factors binding the upstream sequences of *acsp30 *in the S strain as compared to the R strain. Competition binding experiments demonstrated that binding of nuclear proteins for complex formation was highly specific. By using two deletion mutants of the 400 bp region, we were able to narrow down the regions that were important for the assembly of both complexes.

The formation of complex A was seen even in the smallest, 100 bp region used for EMSA experiments but complex B was completely abolished in this region and required an additional 88 bases. Importantly, S strain nuclear extracts lacked or had very low levels of transcription binding factors (TBF) forming complex B. Complex A formation remained unaffected on using nuclear extract from the S strain, indicating that the nuclear proteins forming complex A were present in both the strains but, the sequence difference between R and S upstream sequences was critical for its formation. On the other hand, formation of complex B was sensitive to both the sequence and the presence of transcription binding factors in the nuclear extract. Identification and characterization of these TBFs would be necessary to further evaluate the differences in the two strains. The 2-D analysis of nuclear proteins from S and R strains will probably reveal the identity of the TBFs responsible for high level expression of *acsp30 *transcript and other genes contributing to the refractory phenotype.

## Conclusion

In conclusion, this is the first report of an immune-responsive serine protease from a field-collected refractory strain of *Anopheles culicifacies*, the main vector of malaria in India. Transcript abundance of *acsp30 *in naïve adult refractory mosquitoes, even in the absence of any immune challenge, suggests possibility of its contribution towards refractory phenotype to *A. culicifacies *population. Through a series of EMSA experiments and *in silico *analysis, we have tentatively identified potential regulatory binding motifs that could be responsible for differential expression of *acsp30 *in R and S strains of *A. culicifacies*. Further analysis of factors responsible for conferring refractory phenotype to mosquito against malaria parasite may pave way towards identification of critical intervening steps to abort parasite development in mosquito.

## Methods

### Mosquito rearing

Cyclic colonies of S and R strains of malaria vector, *Anopheles culicifacies*, were reared in an insectary at National Institute of Malaria Research, Delhi maintained at a temperature of 28 ± 1°C and 75 ± 5% relative humidity and fitted with simulated dusk and dawn machine with a photoperiod of 14 hour day and 10 hour night as described by Adak *et al*. (1999) [39].

Mosquitoes were fed upon 1% glucose soaked pads and raisins. Female mosquitoes were offered rabbit blood for ovarian development. Following hatching, larvae were reared in enamel trays containing de-chlorinated water and fed on powdered dog biscuits and brewer's yeast tablets in 3:2 ratios.

### Cloning of *ascp30 *of *A. culicifacies*

Total RNA was isolated from the body tissue of refractory population of *A. culicifacies *by the TRIZOL Reagent method according to the manufacturer's instructions (Invitrogen, USA). To avoid contamination of the digestive proteases, the gut was removed from the mosquitoes aseptically. Up to 5 μg total RNA was used for cDNA synthesis using reverse transcriptase enzyme (Superscript II) and adapter primer (AP, 5' GGC CAC GCG TCG ACT AGT ACT TTT TTT TTT TTT TTT 3').

Degenerate primers, SerPr1 (5'-TGG GTC/G C/GTG ACC/G GCC/G GCG/A/T/C CAC/T-3') and SerPr2 (5'-ACG AGC/G CGA/G CCA/G CCC/G GAA/G TCG/A/T/C-3') were designed based on the sequence of reported serine protease in *A. gambiae *[21]. Using these degenerate primers and cDNA as template, a 450 bp fragment was amplified. The fragment was cloned into pGEM-Teasy vector (Promega Corporation, USA) and sequenced by Microsynth (Switzerland). On the basis of sequence of cloned insert, a set of gene-specific primers, DSerPr1 (5'-CGT GCA CTT GAA CAT TAT CTC-3') and DSerPr2 (5'CTG GTT GGC AGC GTC ACG A-3') were synthesized, to obtain 5' and 3' ends of the gene respectively, by RACE (rapid amplification of cDNA ends) according to the protocol described in the 5' and 3' RACE kit manuals (Invitrogen). Gene-specific end primers, 10FMOS (5'-ATG AAA CTG TTC ATC GTC GT-3') and 11R (5'-TTC AGT ACT TGA TGC CAG ATT-3') were designed and synthesized on the basis of sequences of 5' and 3' RACE fragments to obtain the full-length serine protease gene. Using these end primers and cDNA as template, a complete 816 bp gene (ascp30) was isolated, cloned in pGEMT-easy vector and sequenced. All the primers were synthesized from Microsynth.

### Dissections and RNA extractions

The mosquitoes were dissected on clean new slide in a drop of ice-cold sterile DEPC-treated water and body tissue (abdomen and thorax) was collected by removing midgut, heads, legs and wings. Body tissue from ten mosquitoes was pooled in 500 μl of TRIZOL. Mosquito larvae of the different instars were also collected and stored in TRIZOL at -70°C.

The tissues were ground to homogeneity with a battery-driven hand-held homogenizer using DEPC-treated sterile grinder tips and processed for RNA extraction by TRIZOL Reagent method. Contaminating DNA was removed by treatment with RQ1 DNase (Promega Corporation) according to manufacturer's instructions. The total RNA isolated was used as a template for semi-quantitative and real time RT-PCR analysis of *acsp30 *transcripts using β-actin as internal control.

### Blood feeding strategy

Rodent malaria parasite, *Plasmodium vinckei petteri *279BY, supplied by Prof. Irene Landau, Museum National d'Historie Naturelle, Paris [41], was maintained on rodent-mosquito cycle at the National Institute of Malaria Research, Delhi [7]. Cryo-preserved *Plasmodium vinckei petteri *infected blood isolates (500 μl) were mixed with an equal volume of incomplete RPMI culture media and were inoculated into healthy 4–5 weeks old Balb/c mice. Thin blood smears prepared from peripheral blood of infected mice were fixed in methanol and stained in JSB stain [42]. The slides were examined under oil immersion lens of compound microscope (Carl-Zeiss, Germany) for the presence of various stages of parasite. Ideal time for feeding was ascertained by periodically monitoring the parasitemia of infected mice and looking for the presence of at least 0.5% mature gametocytes. Four- to six-day old mosquitoes were starved by depriving them of raisin and glucose pads for nine hours. Approximately 100–200 starved mosquitoes were held in cages and gametocyte-positive mouse was placed in the cage for 1 hour during daytime for feeding. Subsequently, the full-fed mosquitoes were separated from unfed and partially-fed mosquitoes in a separate cloth cage labeled accordingly and were maintained at 23–24°C temperature and 65–70% relative humidity in the insectary. Mosquitoes were dissected after 6, 12, 18 and 24 hours of blood feeding and the tissues were stored in TRIZOL at -70°C until analysis.

### Semi-quantitative RT-PCR analysis

The relative transcript abundance of *acsp30 *in naïve mosquitoes (S and R strains) was determined by semi-quantitative RT-PCR using a One-step RT-PCR kit (Qiagen GmbH, Germany). The amount of RNA in both the tissues was normalized to the β-actin gene. The sequences of the primer pairs used for amplifying β-actin were β-actinFor (5'-CAG ATC ATG TTT GAG ACC TTC AAC-3') and β-actinRev (5'-GA/C/TC CAT CTC C/TTG CTC GAA A/GTC-3'). Using the normalized amount of RNA as template, *ascp30 *transcript was amplified by using gene-specific primers, forward 10F (5'-ATCAGTTACCAATCGATCTTGCC-3') and reverse 4R (5'-CGTACGTTCCCATGCACTG-3') to obtain a 500 bp amplicon. The amplification regimen was as follows: reverse transcription at 48°C for 30 minutes, inactivation at 95°C for 15 minutes and the PCR at 94°C for 30 seconds, 52°C for 30 seconds and 72°C for 30 seconds for 33 cycles followed by a final extension of 72°C for 10 minutes. Before loading on 1% agarose gel, the RT-PCR products were treated with 1 μg of RNase (Qiagen GmbH) at 37°C for 10 minutes to eliminate template RNA. The gel was photographed with Polaroid 667 black and white print film.

### Real Time RT-PCR analysis

Real time RT-PCR was performed using Quanti Tect SYBR Green RT-PCR kit (Qiagen GmbH) and iCycler TM system (Bio-Rad Laboratories, USA) to measure relative transcript levels of *acsp30 *in naïve R and S mosquitoes, in various developmental stages of R strain mosquito and after blood-feeding and parasite-feeding treatments. The Primer 3 web-based tool was used to design gene-specific primers, ensuring that the length of the PCR product was 300 bp. β-actin amplicon was obtained by using β-actinFor and β-actinRev primers and *acsp30 *amplicon was obtained by using AcSp30For (5'-GTC AGA CCG CTG GTG GTA AT-3') and AcSpRev (5'-CTC ACG GTT GAG GAA CGT CT-3') primers. Each 25 μl reaction mixture contained 2 μl of template RNA (100–500 ng/μl), 2× QuantiTect SYBR Green RT-PCR Master Mix, 2 μl of primers (5 pmoles/μl), RNase-free water and 0.25 μl of QuantiTect RT enzyme Mix. Real-time cycler conditions included a preliminary reverse transcription at 48°C for 30 minutes, an initial activation step at 95°C for 15 minutes and 40 cycles of denaturation (94°C), annealing (52°C) and extension (72°C) for 30 seconds each. The final step included gradual temperature increase from 50°C to 94°C at the rate of 1°C/10 seconds to enable melt-curve data collection. A non-template control (NTC) was run with every assay. The threshold cycles (C_T_) were recorded for *acsp30 *and *β-actin *amplicons during each experiment. Difference between the C_T _of *β-actin *and *acsp30 *or ΔC_T _was determined and the relative abundance of *acsp30 *was calculated in different treatments using Comparative C_T _method using the formula 2^-ΔΔ*CT *^[43].

### Isolation of genomic clone of *acsp30*

Genomic DNA (gDNA) was isolated from the body tissue of R and S strains of mosquito by the method of Henry *et al*., 1990 [40]. Using gDNA as template and end primers, 10FMOS and 11R, the genomic clones of *acsp30 *from both strains were amplified by PCR. The obtained amplicons were cloned into pGEM-Teasy vector, sequenced by ABI PRISM and a comparative analysis of the nucleotide sequence from both strains was done using the ClustalW (MacVector Version 7.0).

### Isolation and cloning of upstream regulatory sequences

Three nested gene-specific reverse primers; R1 (Biotinylated) (5'-ACTACGAGGAGATTCACGCATGG-3'), R2 (5'-TTA TTCACACCTTGAGTTCAATTG-3') and R3 (5'-GGCTAAAACGACGACGATGAACAG-3') were designed from the 5' end of serine protease cDNA sequence. Using 50 ng of genomic DNA as template, PCR was performed in four different tubes with four forward walker primers (WP) [WP1, 5'-CTAATACGACTCACTATAGGGNNNNATGC-3'; WP2, 5'-CTAATACGACTCACTATAGGGNNNNGATC-3'; WP3, 5'-CTAATACGACTCACTATAGGGNNNNTAGC-3'; WP4, 5'-CTAATACGACTCACTATAGGGNNNNCTAG-3'] and the reverse biotinylated primer, R1 according to the directional walking method described by Mishra *et al*., 2002 [44]. Amplified biotinylated products were then immobilized on streptavidin-linked paramagnetic beads and non-biotinylated DNA was washed off. Nested PCR was carried out using the above immobilized PCR product as template and gene-specific primer, R2 and T7-based forward walker primer (5'-CTAATACGACTCACTATAGGG-3'). Amplified PCR products were cloned into pGEM-Te vector and sequenced by Microsynth.

Forward primers containing the *Kpn*I site were designed and synthesized. They were located at 702 bp and 333 bp upstream to the translational start site (ATG) at +1 position. These included; RefDelFor702, (5'-GGTACCCAATGACGCGTTAAGCC-3'), RefDelFor333, (5'-GGTACCGATAAGAACGACCTGG-3') for the refractory strain and SusDelFor702, (5'-GGTACCTCCTGATCAACTATAT-3'), SusDelFor333, (5'-GGTACCGATAAGAACGAC CTG G-3') for the susceptible strain. The reverse primers were designed from a region just upstream to the ATG and contained *Xho*I restriction site. These included, RefSPRev, (5'-CTCGAGGATTAG TACTGACCACTACGG-3') for the R strain and SusSPRev, (5'-CTCGAGGATTAGTACTGACCTCTACGG-3') for the S strain. Using the DNA of the pGEM-Teasy clones as template and the above primers, a 32 cycle PCR was carried out with the following conditions; 94°C for 30 seconds, 52°C for 30 seconds and 72°C for 1 minute with a final extension at 72°C for 5 minutes. PCR products were again cloned into pGEM-Teasy vector. Plasmids prepared from the positive colonies were double digested with *Kpn*I and *Xho*I. The fallout was eluted from the gel using Qiagen gel extraction kit and ligated into promoter-less luciferase reporter vector pGL3 Basic (Promega Corporation), which was pre-digested with *Kpn*I and *Xho*I enzymes. Resulting reporter plasmids were named pGL3-Ref702, pGL3-Sus702, pGL3-Ref333 and pGL3-Sus333.

### Computer-based sequence analysis

The upstream sequences of *acsp30 *isolated from R and S strains were analyzed for potential transcription factor binding sites using the MatInspector program [28]; matrix library was set as Insect library, the core similarity at 0.75 and optimized matrix similarity was used for both the strains. The upstream sequences for the two strains were aligned using the ClustalW program (MacVector Version 7.0) and EMBOSS alignment programs. The core promoter elements and other vertebrate transcription binding factors were identified using reported consensus sequences as described in the results.

### Cell culture, transfections and luciferase assay

*Drosophila *S2 cells were obtained from Invitrogen and were maintained at 27°C in Schneider's *Drosophila *medium (Invitrogen) with 10% heat inactivated fetal bovine serum in 25 cm^2 ^cell culture flasks. One day before transfection, S2 cells were seeded at 0.75 × 10^6 ^cells/ml in 24-well tissue culture plates to achieve 80% confluency. DNA (1.5 μg) was transfected using cellfectin (Invitrogen) according to the manufacturer's instructions. The transfection was carried out in serum-free medium for 8–12 hours. The transfection medium was then replaced with serum-containing medium and the cells were harvested 48 hours after transfection in 50 μl of 1× Cell Culture Lysis Reagent. Luciferase activity was then measured using the Luciferase assay system (Promega Corporation) and the luminescence was read on a Packard LumiCount manual luminometer. A pGL3-Control vector containing luciferase reporter under control of SV40 promoter served as a control. Each transfection was repeated three times and the luciferase activity was measured twice for each sample.

### Preparation of nuclear extracts

Five-day old, adult female mosquitoes were dissected on a clean slide in a drop of ice-cold sterile phosphate-buffered saline (PBS) pH 7.4 under a dissecting microscope. The midgut, head, legs and wings were removed and the body tissue of 100 to 200 mosquitoes from both R and S strains were pooled and stored under liquid nitrogen until required. The tissues were ground to homogeneity in a minimum volume of PBS, with a battery-driven hand-held homogenizer using sterile grinder tips and processed for nuclear protein extraction according to the protocol of Lin *et al*., (2004) [45] with minor modifications. Briefly, homogenized tissue was solubilized in buffer A (10 mM HEPES, 10 mM KCl, 1 mM EDTA, 1 mM dithiothreitol, 0.5 mM phenylmethylsulfonyl fluoride, 0.1 mM Na_3_VO_4_, 10% glycerol and 1 μg/ml of aprotinin, pepstatin, and leupeptin (pH 7.9) for 30 min at 4°C by gentle rocking on a nutator and centrifuged at 12,000 × *g *at 4°C for 1 min. The supernatant containing the cytoplasmic proteins was discarded and the pellet was then extracted with buffer B (20 mM HEPES, 350 mM NaCl, 10 mM KCl, 1 mM EDTA, 1 mM dithiothreitol, 1 mM phenylmethylsulfonyl fluoride, 0.1 mM Na_3_VO_4_, 0.2% Nonidet P-40, 10% glycerol, 1 μg/ml aprotinin, pepstatin and leupeptin, pH 7.9) at 4°C for one hour by gentle rocking on a nutator as described above. The extracts were centrifuged at 13,000 × *g *at 4°C for 5 minutes and the supernatants (nuclear extract) were snap frozen in liquid N_2 _and stored at -70°C for subsequent use in EMSAs. All the protein concentrations were estimated by the method of Bradford (1976) [46].

### Electrophoretic mobility shift assay (EMSA)

Primers were designed to encompass the 400 bp upstream region (-702 to -302 bp) of *acsp30 *that was responsible for difference in promoter strength in the two strains. Three different fragments (100 bp, 188 bp and 400 bp) from upstream regions of R and S strains were selected for EMSAs. The DNA probes for EMSAs were prepared by amplifying and radioactively labeling the fragments by PCR in a 50 μl reaction mixture. The reaction consisted of 10 ng template (upstream regions from R and S), 10 pmol of each primer, 100 μM dNTPs, 10 μl α-^32^P-dCTP (10 mCi/ml, 3000 Ci/mmol, PerkinElmer, USA), and 2.5 units of *Taq *DNA polymerase (Clontech, USA). The PCR included 32 cycles of 30 s denaturation at 94°C, 30 s annealing at 52°C and 30 s extension at 72°C, followed by a final extension of 5 min at 72°C. Labeled probes were purified using a QIAquick nucleotide removal kit (Qiagen GmbH) and the counts were checked by scintillation counter (Beckman, USA).

The probes were named based on the size of the amplified DNA fragment. In the R strain, R100 probe (-702 and -602 bp) was amplified using RefUp702For (5'-CAA TGA CGC GTT AAG CCT GAT-3') and RefUp602Rev (5'-GAT CGC CGT CGT CCA TCA ACA-3') primers; R188 probe (-702 and -514 bp) by RefUp702For and RefUp514Rev (5'-TTC ATG TGG TTT CAT GAT TTA TTA-3') primers; R400 probe (-702 and -302 bp) by RefUp702For and RefUp302Rev (5'-CTT TCG AAT GAC GCC AGG T-3') primers. Similarly for the S strain, S100 probe (-702 and -602 bp) was amplified using SusUp702For (5'-TCC TGA TCA ACT ATA TGG GTT CCT-3') and SusUp602Rev (5'-CTA TTG AAA GAA TCA ATT TGC TAA-3') primers; S188 probe (-702 and -514 bp) by SusUp702For and SusUp514Rev (5'-TTC ATA TCT CAT CAT TTA TTA AAA ATT-3') primers; S400 probe (-702 and -302 bp) by SusUp702For and SusUp302Rev (5'-ATT TCG TAA TGA CGG CCA GGT-3') primers. EMSAs were performed using 10 to 20 μg of nuclear extract from either R or S and incubated at 37°C for 25 minutes with ^32^P-labeled probes (30,000 c.p.m. per reaction) and 2 μ*g *of poly (dI-dC) (Sigma). The binding buffer contained 20 mM HEPES (pH 7.9), 1 mM dithiothreitol, 1 mM EDTA (pH 8.0), 1.5 mM MgCl_2 _and 4% glycerol, in a final volume of 20 μl. For competition experiments, a 100-fold molar excess of unlabeled probe was pre-incubated in the reaction mixture at 37°C for 10 min before labeled probe was added. The DNA-protein complex was resolved on a 2% agarose gel (pre-run for 1 h at 4°C) in 0.5× Tris-borate EDTA (TBE) buffer at 200 V at 4°C. Gels were dried and exposed for autoradiography. Scanning was performed using the Typhoon 9210, Variable Mode Imager from Amersham Biosciences, USA. The intensities of the signals were quantified by the Image Analysis Software, ImageQuant TL (Amersham Biosciences) and represented as the percentage ratio of S to R signal intensities. The accession number of the serine protease gene is [GeneBank:AY995188].

## Authors' contributions

JR carried out real-time PCR, transfection studies in cell culture and EMSAs. NA carried out isolation and cloning of serine protease gene and its upstream regulatory sequences. Both, JR and NA, have also been involved in drafting the manuscript. AS carried out mosquito rearing and blood feeding experiments. PM performed EMSA experiments and gave important input in analysis and interpretation of data. TA provided the susceptible and refractory strains of *A. culicifacies *and was involved in dissections of mosquitoes, collection of body tissue, preparation of nuclear extracts and participated in the design of the study. VSC gave final approval of the version to be published. VSC and RKB conceived of the study, participated in its design and coordination and also revised the manuscript critically. All authors read and approved the final manuscript.

## Figures and Tables

**Table 1:** Transcription binding sequences from the insect family in upstream regions.

**Family/matrix**	**Refractory strain Position (from-to)**	**Susceptible strain Position (from-to)**
Drosophila paired homeodomain	Absent	75–95
Suppresor of Hairless	Absent	139–151
Drosophila paired homeodomain	Absent	159–179
Heat shock factor (Drosophila)	Absent	175–195
Drosophila PAX6 P3 homeodomain binding site	Absent	275–295
Drosophila paired homeodomain	Absent	315–335
Dorsal, protein for dorso-ventral axis formation (c-rel)	Absent	331–341
Hunchback, early maternal and zygotic zinc finger gene	Absent	394–406
Crocodile regulator of head developement	404–416	Absent
Heat shock factor (Drosophila)	529–549	Absent
Hunchback, early maternal and zygotic zinc finger gene	529–541	Absent

**Table 2:** Putative transcription binding sequences in upstream region of refractory and susceptible strains of *A. culicifacies *that are homologous to insect immune responsive genes and vertebrate family.

**Putative binding sequences**	**Refractory strain Position (from-to)**	**Susceptible strain Position (from-to)**
Arthropod initiator	547–551 691–695	547–551 691–695
Hepatic nuclear factor 5	237–242 408–413	237–242 408–413
GATA Factors (1, 2 and 3)	664–676, 356–368, 512–524, 266–278, 366–378, 206–218, 507–519	36–376, 664–676, 511–523, 115–127, 172–184, 201–213, 506–518
Interferon Consensus Response Elements (Icre)	399–405	60–66, 68–74, 181–187, 404–410, 522–528, 532–538
Mammalian Type I-Interleukin Response Element (Nf-I16)	48–56 350–358	399–398 600–608
Nuclear Factor Endothelial Leucocyte Adhesion Molecule (NF-ELAM1)	544–550	544–550
Nf-Kappa B	Absent	Absent
